# Assisted Reproductive Technology as Predictor for Preeclampsia: A Systematic Review of Current Clinical Prediction Models

**DOI:** 10.1007/s43032-026-02168-7

**Published:** 2026-09-15

**Authors:** Géraldine C. M. Lafeber, Marie-Louise P. van der Hoorn, Saskia le Cessie, Eileen E. L. O. Lashley

**Affiliations:** 1 Department of Obstetrics and Gynaecology, Leiden University Medical Center, Albinusdreef 2, 2333 ZG Leiden, the Netherlands; 2 Department of Clinical Epidemiology, Leiden University Medical Center, Albinusdreef 2, 2333 ZG Leiden, the Netherlands; 3 Department of Obstetrics and Gynaecology, Erasmus Medical Center, Molewaterplein 40, 3015 CN Rotterdam, the Netherlands

**Keywords:** Assisted Reproductive Technology, Epidemiology, Preeclampsia, Prediction model, Risk stratification, Systematic review

## Abstract

Assisted Reproductive Technology (ART) increases the risk of preeclampsia (PE), yet no specific prediction model tailored to the ART population exists. This review evaluates prediction models incorporating ART as a predictor, focusing on risk of bias, performance, generalizability and clinical applicability. A systematic MEDLINE search up to July 2023 identified models predicting PE that included ART as a predictor. Exclusion comprised studies on outcomes after PE onset, univariable models and validation studies of previously validated models. Risk of bias, applicability and generalizability were assessed using the Prediction model Risk Of Bias ASsessment Tool (PROBAST) and the Transparent Reporting of a multivariable prediction model for Individual Prognosis and Diagnosis (TRIPOD) guideline. Out of 15,305 records, 161 articles were assessed in full-text, yielding fifteen eligible studies. Eleven studies were added from a prior systematic review. Performance varied widely (AUC 0.58–0.99) and only few (7/26) models had been externally validated. Risk of bias was unclear in most studies (21/26), due to analytical issues. Despite ART being an established risk factor for PE, robust and generalizable models with proven external validity are lacking. Future research should prioritize development of a high quality prediction model including ART, or a model developed specifically within ART populations.

**Supplementary Information:**

The online version contains supplementary material available at https://doi.org/10.1007/s43032-026-02168-7.

## Introduction

Infertility affects 8–12% of couples worldwide. Assisted Reproductive Technology (ART), posing these couples an opportunity to conceive [1], is becoming increasingly successful. Each year, over 3 million ART cycles are reported [1], reflecting an ongoing expansion of indications.

While ART has transformed the landscape of reproductive health, it has also introduced new challenges related to potential adverse obstetric outcomes. Studies indicate that pregnancies resulting from ART have a nearly doubled risk of hypertensive disorders of pregnancy, including preeclampsia (PE) [2–4], compared to naturally conceived pregnancies. PE, characterized by hypertension accompanied by proteinuria, maternal end-organ dysfunction, or uteroplacental dysfunction [5], remains one of the leading causes of maternal mortality when left untreated or not recognized in time [6]. Moreover, hypertensive complications in pregnancy are associated with long-term maternal risks, including increased cardiovascular disease and decreased quality of life [7–9]. Additionally, severe PE increases the risk of fetal growth restriction and preterm birth due to underlying placental insufficiency [10–12], highlighting the need for timely identification and management, especially in ART pregnancies.

Several theories have been proposed to explain the increased risk following ART. The need for ART itself may reflect underlying reproductive characteristics associated with an increased risk of PE, including advanced maternal age, nulliparity after prolonged infertility, and infertility-related diagnoses [3, 4]. Moreover, recent evidence suggest that specific ART related factors, such as programmed frozen embryo transfer cycles lacking a corpus luteum and its vasoactive products, and multiple gestation resulting from multiple embryo transfers, may causally contribute to PE risk [1]. Finally, altered maternal–fetal immune interactions, as reflected in oocyte donation, have been hypothesized to play a role in PE development [8, 10, 12].

While many prediction models for PE exist, none specifically address ART populations [13]. Initially, this review aimed to assess models developed exclusively for ART pregnancies, but their absence required a broader approach. Therefore, this review identifies prediction models for preeclampsia incorporating ART as a predictor and evaluates their risk of bias, performance, generalizability, applicability and reproducibility using TRIPOD and PROBAST.

## Materials and Methods

This study was performed in accordance with the Preferred Reporting Items for Systematic Reviews and Meta-Analyses Statement guidelines [14]. This review is registered in PROSPERO (ID CRD420250477748). Registration was completed in 2025 after the process of study selection. No deviations from the registered protocol were made.

### Search Strategy and Study Selection

A systematic literature search was conducted in MEDLINE, covering the period from January 2017 – July 31 st 2023, to update the search from De Kat et al. (2019) [13]. While De Kat et al. identified general prediction models for PE and selected only models with AUC ≥ 0.6, this review specifically focuses on models that included ART. The search strategy included free-text terms as ‘prediction model’ and ‘preeclampsia’. The complete search string is provided in Supplement 1.

Two researchers (GL and EELOL) independently performed the literature search. Results were exported into a citation management software (Endnote), and articles were subsequently screened by both researchers. The screening process involved two stages: first, titles and abstracts were reviewed; second, full-text articles were analyzed. Any discrepancies between the researchers were resolved through consensus meetings. Additionally, the full-text of the studies included in the review by De Kat et al. [13] were analyzed and incorporated into the final study selection if the prediction models presented included ART as predictor.

### Eligibility Criteria

We included studies that developed (development only) or validated (validation only), or both developed and validated a prediction model for PE, with the definition as used by the authors and with ART as a predictor in the final model. If a validation study of a previously developed model within the inclusion period was available, we included both the development and corresponding validation study.

ART was defined as any fertility-related treatment in which hormonal stimulation takes place [15]. For the current review, studies that reported mode of conception rather than explicitly identifying ART as a predictor were also included. Studies published in English were eligible for inclusion. In the first stage, all prediction models for PE were considered. In the second stage, we determined whether ART was included in the final model. Timing of use of the prediction model was preconceptionally or in early pregnancy.

Exclusion criteria were studies focusing on outcome after PE onset, such as publications on the fullPIERS model [16]. We also excluded studies that focused solely on single variables, such as biomarkers [17], without formulating a prediction model. Furthermore, studies testing the performance of previously externally validated models, such as those from the Fetal Medicine Foundation, were excluded unless they involved adding at least one new variable or extended the original model. Systematic reviews, case reports, guidelines and letters were also excluded.

### Risk of Bias Assessment

The risk of bias assessment for this systematic review was performed using the Prediction model Risk Of Bias ASsessment Tool (PROBAST) [18, 19], a framework specifically designed for evaluating prediction models. PROBAST involves risk of bias assessment across four domains: participants, predictors, outcome and analysis. In addition, it assesses applicability of prediction models. PROBAST does not address confounding, as it is not relevant in prediction studies.

Notably, De Kat et al. [13] employed the Quality in Prognosis Studies (QUIPS) tool for their quality assessment. Although QUIPS evaluates six domains, it was not designed for scoring prediction models. Accordingly, the studies included from De Kat et al. [13] were also assessed on bias using PROBAST.

Risk of bias was rated low (+), unclear (?) or high (-). The overall rating reflects an aggregate assessment across all domains. For example, a study rated as “?” in the Analysis domain was still classified as overall “ + ” when the remaining domains were all rated “ + ”. Conversely, a study with “?” in the Analysis domain was rated overall “-” when the other domains were consistently “-”.

For the PROBAST tool, studies were classified having a low risk of bias if appropriate data sources, such as cohort studies were used, and included participants who corresponded to unselected participants representative of the target population. Predictors were required to have clear and consistent definitions, assessed uniformly across all patients. Continuous and categorical predictors had to be handled appropriately, by avoiding data-driven categorization and properly addressing non-linearity. Outcome information should not have been used during predictor assessment, and all predictors should have been available at the time the model was intended to be used. Outcomes were determined objectively, following established guidelines or prior publications on the topic and were assessed uniformly across all participants without knowledge of predictor information. The time interval between predictor assessment and outcome determination needed to be appropriate to ensure the recording of relevant and representative outcomes. Studies were also required to have an adequate sample size, with at least 20 events per variable and continuous variables could not be categorized into two or more groups when included in the model. Missing data for predictors or outcomes had to be appropriately handled, either through explicit reporting of minimal missing data or the application of multiple imputation. Predictor selection based solely on univariable analysis was considered inappropriate. Complexities in the data, such as non-linearity or interactions, had to be identified and addressed, and model performance was required to be evaluated using metrics like calibration and discrimination. Internal validation techniques, including bootstrapping or cross-validation, were expected, with adjustments for optimism or overfitting. Finally, predictors and their assigned weights in the final model needed to align with the results of reported multivariable analysis. Validation could not proceed if any predictor data required by the model are missing, as this compromises the assessment of the model's performance and reliability.

Applicability was rated as low (+), unclear (?) or high (-) concern regarding the relevance of the model to the review question. Judgements were based on whether the included participants and setting matched the review question, whether the definition, assessment or timing of predictors in the model match the review question, and whether the outcome, its definition, timing or determination match the review question. Detailed overview of domain specific assessments and derivation of the overall ratings are provided in Supplement 2.

Risk of bias and applicability were independently assessed by GL and EELOL. Any discrepancies in scoring were discussed with a third observer (MLvdH) in a consensus meeting.

### Assessment of Reporting Quality

The quality of reporting in the prediction models was assessed using the TRIPOD statement [20], which includes a checklist of 22 items and 15 subitems focused on key reporting aspects of title/abstract, introduction, methods, results and discussion. GL and EELOL assessed each study, marking each item as ‘yes’ if it was sufficiently reported or ‘no’ if it was not. Any discrepancies in scoring were discussed with a third observer (MLvdH) in a consensus meeting. A reporting quality score was calculated based on the number of items that were met. Although this score is not a validated measure for prediction models, it has been used in other studies to summarize reporting quality [21–23].

### Data Extraction

GL extracted data from all selected articles, using the Checklist for Appraisal and data extraction for systematic Reviews on prediction Modelling Studies [24]. Data extraction comprised study design, participants, predictors and outcome, the timepoint at which the prediction model is intended to be applied, model development methods, model performance and validation statistics, and means for clinical application. In studies with multiple models, only those with ART in the final predictor selection were extracted. Additionally, the impact of ART and its significance to the model's accuracy and predictive power were assessed. Data were synthesized narratively and presented in tables.

### Model Performance

As model performance measures, metrics of discrimination and calibration were extracted. Discrimination measures the model’s ability to distinguish between patients who experienced the outcome and those who did not, typically assessed using the concordance statistic (c-statistic), which represents the area under the receiver operating characteristic (ROC) curve. A c-statistic of 1 indicates perfect discrimination, while a value of 0.5 indicates no better than random guessing. Calibration assesses the agreement between predicted probabilities and observed outcome frequencies, often visualized with a calibration plot that compares observed frequencies against predicted probabilities [25]. In case c-statistics and calibration slopes were not provided, detection rates (DR) and false positive rates (FR) were reported.

## Results

### Study Selection

**Fig. 1:**
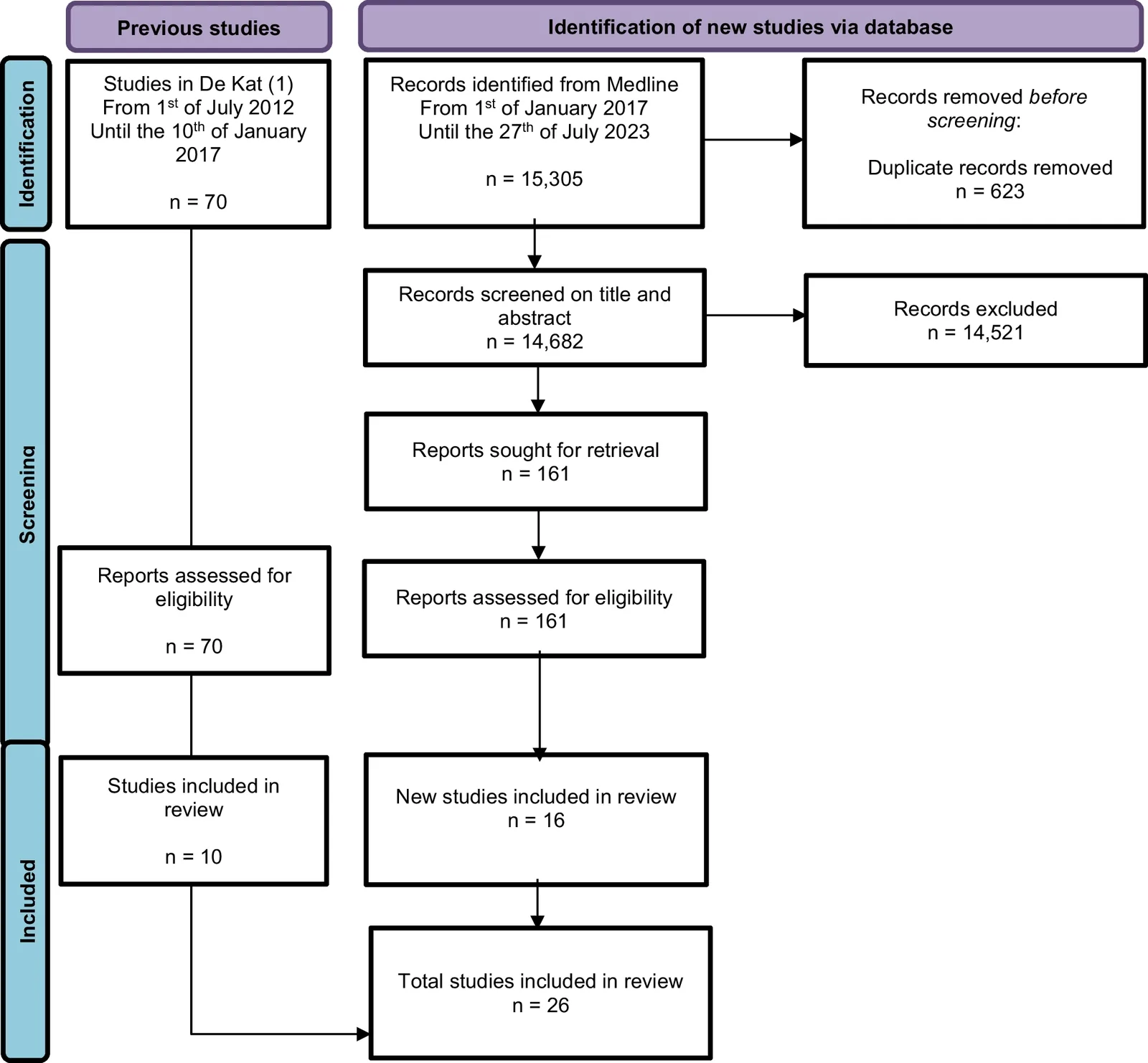
Preferred reporting items for systematic reviews and meta-analyses flowchart

The study selection process is illustrated according to the Preferred Reporting Items for Systematic Reviews and Meta-analyses (PRISMA) flowchart [26] (Fig. 1). The initial search yielded 15,305 records. After removing duplicates, 14,682 articles advanced to the first screening stage, where titles and abstract were assessed. This resulted in the exclusion of 14,521 articles, leaving 161 for full-text assessment in the second stage. Some studies proposed a model using Bayesian analysis [27] with prior risks based on ‘prior risk factors’ or ‘maternal factors’, though these factors were not always specified [28–32]. Following correspondence with the authors, studies in which ART was not considered a prior ‘maternal factor’, or where authors did not respond, were excluded [28–30]. Ultimately, 16 studies from this search met the eligibility criteria [31–46].

Additionally, full-text screening of the 70 studies included in the research by De Kat et al. [13] identified 10 extra studies with ART defined as predictor [47–56]. Consequently, a total of 26 articles were included in the current review.

### Study Characteristics, Sample Size and Modelling Method

Studies were published between 2009 and 2023. Eleven studies were conducted across multiple centers [31, 34, 35, 39, 41, 45, 49, 52, 53, 55, 56]. The number of participants ranged from 94 [43] to 141.299 [42], with in total 625.001 participants of whom 20.142 developed PE. None of the studies performed a sample size calculation prior to model development. The majority of studies was conducted in Europe [32, 34, 35, 37, 38, 41–44, 47–56], six studies in Asia [31, 33, 36, 45, 46, 57] and one in North America [39]. Key elements of the included studies are summarized in Tables 1 and 2, which distinguish between Bayesian and frequentist models. In prediction model development and validation, frequentist methods estimate parameters based on observations only, assuming true parameters are fixed but unknown. These methods use metrics like confidence intervals and p-values. In contrast, Bayesian methods incorporate prior knowledge, updating beliefs as new data become available, resulting in probability distributions for model parameters [27].

**Table 1:** Summary of the key elements of the studies using Bayes’ theorem combining the *a-priori* risk from maternal factors with biomarkers

Author Year Country	Outcome	Population	Exclusion criteria	Study design	Timing (GA)	Study size	Candidate predictors	Predictors final model	Model type	Final model performance
Poon [47] 2009 UK	GH^‰^; DBP ≥ 90 mmHg in ≥ 4 h, GA ≥ 20; PE^‰^; GH^‰^ with proteinuria ≥ 300 mg/24 h or ≥ 2 + on dipstick	Singleton, attending first routine screening	Missing data, major fetal defect, aneuploidy, fetal death, miscarriage < GA24, termination for social reasons	Cohort	11–14	8,366 of which 165 with PE	Log maternal factor-derived a priori risk for early/late PE, GH (developed in [53])	logMaternal factors, logMAP MoM, log Uterine Artery L-PI MoM	Development only Multivariate logistic regression	Early PE AUC = 0.7 Late PE AUC = 0.80
Wright [48] 2012 UK	GA at delivery with PE^¥^ (preterm PE^¥^ (GA < 34), late-onset PE^¥^ (GA > 34, < 42))	Singleton, attending first routine, phenotypical normal, livebirth or stillbirth > GA24	Aneuploidy, major fetal abnormalities, those ending in termination, miscarriage, fetal death < GA2A, SGA	Cohort	11–13	58,884 of which 1,426 with PE	Maternal factors, UtA-PI, MAP	PE < 34wks Maternal factors Maternal factors, UtA-PI Maternal factors, MAP Combined testing PE < 37wks Maternal factors Maternal factors, UtA-PI Maternal factors, MAP Combined testing PE < 42wks Maternal factors Maternal factors, UtA-PI Maternal factors, MAP Combined testing	Development only Competing risk	PE < 34wks (FPR 10%) DR = 50.5 DR = 75.2 DR = 72.9 DR = 89.7 PE < 37wks (FPR 10%) DR = 43.3 DR = 55.1 DR = 59.3 DR = 71.5 PE < 42wks (FPR 10%) DR = 40.3 DR = 42.2 DR = 53.5 DR = 56.6
Khalil [49] 2012 UK	PE^¥^, preterm PE^¥^ (GA < 34), late-onset PE^¥^ (GA > 34, < 42)	Singleton, attending first routine screening	Missing data, major fetal defect, aneuploidy, fetal death, miscarriage < GA24, termination for social reasons	Cohort multicenter	11^0^–13^6^	7,084 of which 181 with PE	Maternal factors, vascular derived risk, UtA-PI, PAPP-A	Maternal factors Maternal factors, vascular derived risk Maternal factors, vascular derived risk, UtA-PI, PAPP-A	Development only Multivariate logistic regression	AUC = 0.80 AUC = 0.84 AUC = 0.85
Park [50] 2013 UK	GH^¥^, PE^¥^ (early/late); > 140/90 2 × with ≥ 300 mg/24 h or ≥ 30 mg protein/mmol creatinin	Attending first trimester aneuploidy screening	Multiple pregnancies	Cohort	11–13	3,014 of which 83 with PE	Maternal factors (ethnicity, history of chronic hypertension, parity with/without PE, mode of conception), MAP Mom, UtA-L-PI MoM, PAPP-A MoM	Maternal factors (ethnicity, history of chronic hypertension, parity with/without PE, mode of conception), MAP MoM, UtA-L-PI MoM, PAPP-A MoM	Validation only [47]	Early PE AUC = 0.93 Late PE AUC 0.76
Skråstad [51] 2015 Norway	PE^¶^; (SBP ≥ 140 mmHg and/or DBP ≥ 90 mmHg in > 1x ≥ 4–6 h, GA ≥ 20, with proteinuria (≥ 300 mg/24 h or ≥ 2 + on dipstick) Preterm PE^¶^; < GA 37^0^	Nulliparous	GA outside range for Perkin Elmer risk calculation, UtAPI outside range for Perkin Elmer MoM values, if not possible to draw blood, delayed miscarriages, multiple pregnancies, suspected congenital anomalies, stillborn	Cohort	11^0^–13^6^	560 of which 21 with PE	FMF Maternal characteristics, MoM-values of the mean of left and right UtAPI, MAP PAPP-A and PlGF; Perkin Elmer (PREDICTOR) Prior risk (based on BMI, ethnicity, parity, family history of preeclampsia, chronic hypertension and MAP) Posterior risk (based on prior risk and MoMs of MAP, UtAPI, PlGF and PAPPA)	FMF [48] Maternal characteristics, MoM-values of the mean of left and right UtAPI, MAP PAPP-A and PlGF; PREDICTOR [58] ‘Maternal characteristics’, MAP, UtA-PI, PlGF, PAPP-A	Validation only [48, 58] Multivariate logistic regression	PE < GA42: AUC = 0.77 PE < GA37: AUC = 0.94
O’Gorman [52] 2016 UK	Gestational age at delivery with PE PE; BP ≥ 140/90, ≥ 2x, > GA20 with proteinuria ≥ 300 mg/24 h or ≥ 2 + on dipstick	Attending first routine visit, singleton, phenotypically normal birth ≥ GA24, preexisting hypertension, pregnancy-associated hypertension	Aneuploidy, major fetal abnormalities, termination, miscarriage, fetal death < GA24	Cohort multicenter	11^0^–13^6^	35,948 of which 1,058 with PE	Maternal factors, MAP, UtA-PI, PAPP-A, PlGF	MF [58] MF, MAP MF, UtA-PI MF, PAPP-A MF, PlGF MF, MAP, UtA-PI MF, MAP, PAPP-A MF, MAP, PlGF MF, UtA-PI, PAPP-A MF, UtA-PI, PlGF MF, PlGF, PAPP-A MF, MAP, UtA-PI, PAPP-A MF, MAP, PAPP-A, PlGF MF, MAP, UtA-PI, PlGF MF, UtA-PI, PAPP-A, PlGF MF, MAP, UtA-PI, PAPP-A, PlGF	Validation only [58] Multivariable Gaussian distribution 5 × cross validation	AUC = 0.80 AUC = 0.845 AUC = 0.841 AUC = 0.822 AUC = 0.872 AUC = 0.876 AUC = 0.860 AUC = 0.896 AUC = 0.851 AUC = 0.884 AUC = 0.873 AUC = 0.884 AUC = 0.897 AUC = 0.906 AUC = 0.885 AUC = 0.907
O’Gorman [53] 2017 UK, Spain, Belgium, Greece, Italy	PE GH^¥^, PE^¥^ (early/late); BP ≥ 140/90, ≥ 2x, > GA20 with proteinuria ≥ 300 mg/24 h or ≥ 2 + on dipstick	Singleton, maternal age ≥ 18, no serious mental illness or learning difficulty, live fetus on ultrasound GA11^0^−13^6^ and delivery of phenotypically normal live birth or still birth ≥ GA24	Multiple pregnancies, aneuploidy, major fetal abnormality, termination or miscarriage	Cohort multicenter	11^0^–13^6^	9,014 of which 239 with PE	Maternal factors (MF), MAP, UtA-PI, PAPP-A, PlGF	MF [54] MF, MAP MF, UtA-PI MF, PAPP-A MF, MAP, UtA-PI MF, MAP, PAPP-A MF, MAP, PlGF MF, UtA-PI, PAPP-A MF, UtA-PI, PlGF MF, PlGF, PAPP-A MF, MAP, UtI-PI, PAPP-A MF, MAP, PAPP-A, PlGF MF, MAP, UtA-PI, PlGF MF, UtA-PI, PAPP-A, PlGF MF, MAP, UtA-PI, PAPP-A, PlGF	Validation only [55]	AUC = 0.9071 AUC = 0.9309 AUC = 0.8546 AUC = 0.9506 AUC = 0.9667 AUC = 0.9133 AUC = 0.9674 AUC = 0.9339 AUC = 0.9772 AUC = 0.9510 AUC = 0.9644 AUC = 0.9672 AUC = 0.9870 AUC = 0.9769 AUC = 0.9865
Maymon [33] 2017 Israël	PE^&^; ≥ 2 × SBP ≥ 140 mmHg and DBP ≥ 90 mmHg in ≥ 4 h, GA ≥ 20, with proteinuria ≥ 300 mg/24 h or 2 + on dipstick	Twins, age ≥ 18,	Major fetal anomaly, nuchal translucency ≥ 3.5 mm, fetal reduction of high order multiplicity, chromosomal abnormalities, any maternal disease that could affect fetal growth, treatment with LMWH due to thrombophilia	Cohort	16–20	96 twins, of which 9 with PE	‘prior risk factors’^ε^, PAPP-A, PlGF, MAP, UtA-PI, PP13	log_10_PP13, log_10_PAPP-A, log_10_PlGF, log_10_UtA-PI, log_10_MAP, maternal age, BMI, parity, HT in previous pregnancy, (no) ART	Development only	AUC = 0.981 DR = 75% for 10% FPR
Francisco [35] 2017 UK	GA of delivery with PE^¥^ (BP ≥ 140/90, ≥ 2x, > GA20 with proteinuria ≥ 300 mg/24 h or ≥ 2 + on dipstick)	Twins, attendance first routine visit, delivery of phenotypically normal live birth or still birth ≥ GA24	Aneuploidy, major fetal abnormalities, termination, miscarriage, fetal death < GA24, interval of > 3 d between death of one fetus and live birth od second fetus	Cohort multicenter	11^0^–13^6^	95,516 DC, MC and singletons, of which 2,333 with PE	‘Maternal characteristics’ [55] (age, weight, height, racial origin, interpregnancy interval, GA at delivery of previous pregnancy with and without PE, method of conception, chronic hypertension, DM, SLE/APS, DC and MC twins)	‘Maternal characteristics’ [55] (age, weight, height, racial origin, interpregnancy interval, GA at delivery of previous pregnancy with and without PE, method of conception, chronic hypertension, DM, SLE/APS, DC and MC twins)	Development only Competing risk model/Gaussian distribution	Risk cut-off 1/10: Singleton SPR: 0.8 DR PE < 37: 16.1% DR PE > 37: 12.7%
Francisco [34] 2017 UK	GA of delivery with PE^¥^ (BP ≥ 140/90, ≥ 2x, > GA20 with proteinuria ≥ 300 mg/24 h or ≥ 2 + on dipstick) PE^¥^ < GA32 PE^¥^ < GA37 PE^¥^ < GA42	Singletons, twins, attendance first routine visit	Aneuploidy, major fetal abnormality, terminated pregnancies, miscarriage, fetal death < 24 weeks, > 3 days between death fetus 1 and birth fetus 2	Cohort multicenter	11^0^–13^6^	37,048 singletons and twins of which 1,151 PE	Maternal factors, MAP, UtA-PI, PlGF, PAPP-A	PE < 32 wks MF MF, MAP MF, UtA-PI MF, PlGF MF, PAPP-A MF, MAP, UtA-PI, PlGF MF, MAP, UtA-PI, PlGF* MF, MAP, UtA-PI, PlGF, PAPP-A PE < 37 wks MF MF, MAP MF, UtA-PI MF, PlGF MF, PAPP-A MF, MAP, UtA-PI, PlGF MF, MAP, UtA-PI, PlGF* MF, MAP, UtA-PI, PlGF, PAPP-A PE < 42 wks MF MF, MAP MF, UtA-PI MF, PlGF MF, PAPP-A MF, MAP, UtA-PI, PlGF MF, MAP, UtA-PI, PlGF* MF, MAP, UtA-PI, PlGF, PAPP-A	Development and validation [35, 55] Competing risk model;	PE < 32 wks AUC = 0.68 AUC = 0.83 AUC = 0.84 AUC = 0.81 AUC = 0.69 AUC = 0.93 AUC = 0.94 AUC = 0.92 PE < 37 wks AUC = 0.70 AUC = 0.77 AUC = 0.74 AUC = 0.74 AUC = 0.71 AUC = 0.81 AUC = 0.82 AUC = 0.81 PE < 42 wks AUC = 0.69 AUC = 0.75 AUC = 0.72 AUC = 0.71 AUC = 0.71 AUC = 0.78 AUC = 0.79 AUC = 0.78
Sapantzoglou [32] 2021 UK	PE^€^ GA < 37, PE^€^ GA ≥ 37	Singleton, non-malformed liveborn or stillborn neonate	GH, aneuploidy, major fetal abnormality	Cohort	19^1^–23^3^	2,853, of which 76 with PE	‘Maternal factors’ (gestational age, weight, race, method of conception, medical conditions, obstetric history, characteristics of measurement), PSV ratio, MAP, UtA-PI, PlGF	PE < 37wks MF MF, PSVR MF, MAP MF, UtA-PI MF, PlGF MF, sFlt-1 MF, MAP, UtA-PI MF, MAP, UtA-PI, PlGF MF, MAP, UtA-PI, PlGF, sFlt-1 MF, MAP, PSVR MF, UtA-PI, PSVR MF, PlGF, PSVR MF, sFLt-1, PSVR MF, MAP, UtA-PI, PSVR MF, MAP, UtA-PI, PlGF, PSVR MF, MAP, UtA-PI, PlGF, sFlt-1, PSVR PE > 37wks MF MF, PSVR MF, MAP MF, UtA-PI MF, PlGF MF, sFlt-1 MF, MAP, UtA-PI MF, MAP, UtA-PI, PlGF MF, MAP, UtA-PI, PlGF, sFlt-1 MF, MAP, PSVR MF, UtA-PI, PSVR MF, PlGF, PSVR MF, sFLt-1, PSVR MF, MAP, UtA-PI, PSVR MF, MAP, UtA-PI, PlGF, PSVR MF, MAP, UtA-PI, PlGF, sFlt-1, PSVR	Development only Multivariate linear regression Competing risk Posterior distribution of GA of delivery with PE	PE < 37wks AUC = 0.86 AUC = 0.90 AUC = 0.88 AUC = 0.80 AUC = 0.96 AUC = 0.87 AUC = 0.82 AUC = 0.91 AUC = 0.92 AUC = 0.91 AUC = 0.87 AUC = 0.97 AUC = 0.90 AUC = 0.88 AUC = 0.95 AUC = 0.96 PE > 37wks AUC = 0.76 AUC = 0.82 AUC = 0.82 AUC = 0.73 AUC = 0.79 AUC = 0.76 AUC = 0.80 AUC = 0.81 AUC = 0.81 AUC = 0.86 AUC = 0.81 AUC = 0.84 AUC = 0.82 AUC = 0.89 AUC = 0.86 AUC = 0.86
Chaiyasit [31] 2022 China, Thailand, Singapore	PE^¥^, preterm PE^¥^ (GA < 34), late-onset PE^¥^ (GA > 34, < 42)	Singleton	Unspecified	Cohort multicenter	11–13	7,877 of which 141 with PE	‘Maternal factors^ε^’, PlGF,sFlt-1, MAP, SBP	Any-PE IPPIC: Clinical, SBP IPPIC: Clinical, SBP, PlGF, sFLt-1 CR approach: MF CR approach: MF, MAP CR approach: MF, PlGF Early-PE IPPIC: Clinical, SBP IPPIC: Clinical, SBP, PlGF, sFLt-1 CR approach: MF CR approach: MF, MAP CR approach: MF, PlGF Late-PE IPPIC: Clinical, SBP IPPIC: Clinical, SBP, PlGF, sFLt-1 CR approach: MF CR approach: MF, MAP CR approach: MF, PlGF	Delvelopment and validation Regression analysis	PE > 37wks AUC = 0.70 AUC = 0.70 AUC = 0,70 AUC = 0.75 AUC = 0.79 PE > 37wks AUC = 0.60 AUC = 0.82 AUC = 0.77 AUC = 0.77 AUC = 8.85 PE > 37wks AUC = 0.69 AUC = 0.67 AUC = 0.69 AUC = 0.75 AUC = 0.79
Moungmaithong [36] 2023 China	PE^@^ GA < 37, PE GA ≥ 37	Singleton, livebirth ≥ GA24	Aspirin use	Case–control	11–13	1,786 of which 202 with PE	Maternal factors (MF), GlyFn, MAP, UtA-PI, PlGF	MF MF, GlyFn MF, MAP, GlyFn MF, UtA-PI, GlyFn MF, PlGF, GlyFn MF, MAP, UtA-PI, PlGF MF, MAP, UtA-PI, GlyFn MF, MAP, PlGF, GlyFn MF, MAP, UtA-PI, PlGF, GlyFn	Development and validation	AUC = 0.719 AUC = 0.814 AUC = 0.842 AUC = 0.833 AUC = 0.869 AUC = 0.859 AUC = 0.866 AUC = 0.897 AUC = 0.896

**Table 2:** Summary of the key elements of studies providing frequentist models

Author Year Country	Outcome	Population	Exclusion criteria	Study design	Timing (GA)	Study size	Candidate predictors	Predictors final model	Model type	Final model performance
Poon [54] 2010 UK	GH^‰^; DBP ≥ 90 mmHg in ≥ 4 h, GA ≥ 20; PE^‰^; GH^‰^ with proteinuria ≥ 300 mg/24 h or ≥ 2 + on dipstick Early PE; ≤ GA34	Attending first routine screening	Missing data, major fetal defect, aneuploidy, fetal death, miscarriage < GA24, termination for social reasons	Cohort	11^0^–13^6^	8,366 of which 126 with PE	Maternal characteristics Incl. age, ethnicity, smoking, history, medication, parity, method of conception, family history of PE, MAP UtA PI	Early PE Ethnicity, chronic hypertension, previous PE, parity, method of conception	Development only Multivariate logistic regression	AUC = 0.95
Wright [55] 2015 UK	Gestational age in weeks at time of delivery PE GH^¥^, PE^¥^ (early/late); > 140/90 2 × with ≥ 300 mg/24 h or ≥ 30 mg protein/mmol creatinine	Singleton, attending first routine, normal livebirth or stillbirth GA > 24	Aneuploidy, major fetal abnormality, termination, miscarriage or fetal death < GA24	Cohort multicenter	11^0^–13^6^	120,492 of which 2704 with PE	Age, weight, height, BMI, GA, race, chronic hypertension, SLE/APS, smoking, family history of PE, parity, mode of conception	Age, height, race, SLE/APS, IVF, prior PE, previous GA at delivery, chronic hypertension, DM, family history of PE	Development only Gaussian model, 5 × cross validation	All PE AUC = 0.7562 PE < GA37 AUC = 0.7920 PE < GA34 AUC = 0.8106
Perales [56] 2017 Spain	Early-onset PE (SBP ≥ 140 mmHg and/or DBP ≥ 90 mmHg with proteinuria > GA20) < GA34^0^	Women at risk for PE^η^	Hypertension combined with proteinuria, major fetal malformations, chromosome disorders	Cohort multicenter	19–20, 23–24, 27–28	729, of which 24 early-onset PE	sFlt-1/PlGF ratio, PlGF, UtA-PI, MAP, parity, previous PE, ART	1. MAP, multiparity, previous PE, UtA-PI, ART, sFlt/PlGF ratio 2. MAP, multiparity, previous PE, UtA-PI, ART, sFlt 3. MAP, multiparity, previous PE, UtA-PI, ART, PlGF 4. MAP, multiparity, previous PE, ART, sFlt/PlGF ratio 5. MAP, multiparity, previous PE, ART, sFlt 6. MAP, multiparity, previous PE, ART, PlGF	Development only Logistic regression with Lassoo penalization	GA20 1. AUC = 0.87, 2. AUC = 0.83, 3. AUC = 0.83, 4. AUC = 0.87, 5. AUC = 0.82, 6. AUC = 0.82 GA24 1. AUC = 0.92, 2. AUC = 0.90, 3. AUC = 0.89, 4. AUC = 0.91, 5. AUC = 0.88, 6. AUC = 0.85 GA28 1. AUC = 0.91, 2. AUC = 0.88, 3. AUC = 0.86, 4. AUC = 0.92, 5. AUC = 0.91, 6. AUC = 0.89
Sandström[37] 2019 Sweden	PE (overall/early < GA34/preterm < GA 37/term ≥ GA37) SBP ≥ 140 mmHg and/or DBP ≥ 90 mmHg ≥ 2 × with interval of ≥ 4 h, combined with ≥ 300 mg/24 h or ≥ 2 + on dipstick	Singleton, livebirth, nulliparous, delivery ≥ GA 22	Multifetal gestation, absence of information on gestational length, absence of notion of BP ≥ GA15, major fetal malformations, use of aspirin	Cohort	Not specified	62,562 of which 2,773 with PE	Gestational length first examination, maternal age, BMI, MAP, capillary glucose, protein in urine, Hb, previous miscarriage, previous ectopic pregnancy, infertility duration, family situation, region of birth, intoxications^ζ^, FH of PE, FH of HT, infertility (method of conception), cardiovascular disease, endocrine disease, pre-existing diabetes, thrombosis, psychiatric disease, SLE, epilepsy, chronic HT, Crohn/Ulcerous colitis, lung disease/asthma, CKD, hepatitis, gynaecological disease or operation, recurrent UTIs, blood group	Prespecified variables model (FMF/MAP) [55] Family history of PE, country of birth, method of conception, gestational length at registration, maternal age, height, weight, smoking habits in early pregnancy, pre-existing diabetes, chronic hypertension, SLE, MAP Backward selection model Maternal age, BMI, MAP, proteinuria, infertility treatment, diabetes, blood group, alcohol consumption at registration, gestational length at registration, capillary glucose, Hb, infertility duration, FH of PE, FH of HT, CKD, family situation, intoxications^ζ^, region of birth, hepatitis, Mb Crohn/Ulcerous colitis, psychiatric disease Random forest model All candidate predictors	Development only Prespecified variables model, backward selection, random forest	Prespecified variables model (FMF/MAP) [55] AUC = 0.67–0.68, sensitivity for 10% FPR = 28.1–30.6 Backward selection model AUC = 0.66–0.67, sensitivity for 10% FPR = 25.8–28.2 Random forest model AUC = 0.58–0.61, sensitivity for 10% FPR = 18.5–24.3
Lewandowska [38] 2020 Poland	GH^¤^; ≥ 2 × SBP ≥ 140 mmHg and DBP ≥ 90 mmHg in ≥ 4 h, GA ≥ 20 PE^¤^; GH + one organ system disorder (such as proteinuria ≥ 0.3 g/L)	Caucasian, 18–45 years, singleton	Chronic diseases, especially hypertension and DM, kidney and/or liver disfunction, immunological and inflammatory diseases and thromboembolism, fetal defects and birth of phenotypically abnormal child ≤ GA25	Cohort	10–14	7912 of which 24 with PE	Maternal age (continuous, categorical), n previous deliveries, primiparity, interpregnancy interval, pre-pregnancy BMI (continuous, categorical), height, pre-pregnancy weight, GWG (continuous, categorical), prior PE/GH, infertility treatment, IVF, hypothyroidism, no supplementation (folic acid, multivitamins), family history of hypertension (continuous, categorical), ducation, financial status, place of residence	GH 1. Maternal age, primiparity, treatment of infertility 2. Maternal age, primiparity, in vitro fertilization PE 3. Maternal age, primiparity, treatment of infertility	Development only Multifactor regression with forward selection	1. AUC = 0.601 2. AUC = 0.6 3. AUC = 0.585
Boutin [39] 2021 Canada	Preterm PE^*^; hypertension GA ≥ 20 with proteinuria or adverse condition or severe complication (GA < 37)	Nulliparous, singleton, living fetus	Fetal demise, multifetal gestation, multiparous, medical termination, fetal anomalies, aspirin use, CRL < 45 mm, CRL > 85 mm	Cohort multicenter	11^0^–13^6^	4,659 of which 29 with PE	Maternal age, ethnicity, weight, height fertility treatment^α^, chronic diseases^γ^, blood pressure, CRL, UtA-PI, PlGF, sFlt-1, PAPP-A, free β-HCG, AFP	Full model Maternal age, ethnicity, smoking, fertility treatment^α^, body mass index, MoM MAP^δ^, log_10_PAPP-A^δ^, log_10_PlGF^δ^, log_10_sFlt-1^δ^, log_10_free β-HCG^δ^, log_10_AFP^δ^, log_10_UtA-PI^δ^	Development only Multivariable proportional hazard model	Full model AUC = 0.85 (95%CI, 0.48–1.00)
Li [40] 2021 China	PE; GH (≥ 2 × SBP ≥ 140 mmHg and DBP ≥ 90 mmHg in ≥ 4 h, GA ≥ 20), with proteinuria ≥ 300 mg/24 h or 2 + on dipstick Severe PE; GH with new-onset proteinuria, organ dysfunction or uteroplacental dysfunction Later-onset PE, delivery ≥ GA34; Preterm PE, delivery < GA37; Term PE, delivery ≥ GA37	No chronic hypertension/hypertension GA ≤ 20, complete data and delivery in Xinhua hospital Chongming branch	Termination ≤ GA24, major fetal malformations; aspirin use during early pregnancy, eclampsia, placental abruption or HELLP	Case control	Not specified	3,759 of which 191 with PE	age, BMI, mean BP, maternal AC, gravidity, parity, PE in previous pregnancy, prior cesarean delivery, interval ≥ 10 years, nulliparity, multifetal gestations, ART, pre-gestational diabetes, heart disease, thyroid disease, renal disease, auto-immune disease, mental disorder, uterine leiomyoma, adenomyosis, uterine malformations, history of seizure disorder, family history of hypertension; Hb, WBC, platelet counts, direct bilirubin, total bilirubin, ALT, GGT, total protein, albumin, globulin, FPG, TBA, creatinine, serum UN, serum UA	All feature Logistic regression Random forest Extreme gradient boosting Support vector machine Selected features Logistic regression Random forest Extreme gradient boosting Support vector machine Questionnaire-based features Logistic regression Random forest Extreme gradient boosting Support vector machine	Development and validation	All feature AUC = 0.86 AUC = 0.99 AUC = 0.98 AUC = 0.96 Selected features AUC = 0.90 AUC = 0.90 AUC = 0.94 AUC = 0.92 Questionnaire-based features AUC = 0.81 AUC = 0.88 AUC = 0.86 AUC = 0.90
Pétursdóttir Maack [41] 2022 Sweden	PE (≥ 2 × SBP ≥ 140 mmHg and DBP ≥ 90 mmHg in ≥ 6 h, GA ≥ 20, with proteinuria ≥ 300 mg/24 h or ≥ 2 + on dipstick	WC measurement	Miscarriage, termination of pregnancy < GA20, multiple pregnancy, missing data on BMI in early pregnancy, previous inclusion	Cohort (population wide)	GA9.1 (± 2.2)	4,696 of which 209 with PE	Maternal age, parity, smoking, county of origin, chronic hypertension, prepregnancy diabetes, SLE, CKD, history of PE, conception with ovarian stimulation, conception with IVF, MAP, BMI, WC	Prediction model (maternal age, parity, smoking, county of origin, chronic hypertension, prepregnancy diabetes, SLE, CKD, history of PE, conception with ovarian stimulation, conception with IVF, MAP), BMI; Prediction model, WC; Prediction model, BMI, WC;	Development only Logistic regression	AUC = 0.738 AUC = 0.739 AUC = 0.739
Nurkkala [42] 2023 Finland	Preeclampsia, gestational hypertension	Giving birth	Not specified	Cohort Multicenter (national hospital biobanks)	Not known	141,299 of which 6643 with PE	Polygenic risk score (PRS), age, obesity, hypertension, diabetes, gestational diabetes, multifetal pregnancy, in-vitro fertilization pregnancies, renal failure	Clinical model (age, obesity, hypertension, diabetes, gestational diabetes, multifetal pregnancy, in-vitro fertilization pregnancies, renal failure) Clinical model, preeclampsia PRS Clinical model, blood pressure PRS	Development only Logistic regression	c-statistic = 0.634 c-statistic = 0.649 c-statistic = 0.661
Hromadnikova [43] 2023 Czech Republic	HELLP^$^, with or without PE	HELLP cases, healthy controls	Unspecified	Case–control	10–13	94 of which 14 with HELLP	Unspecified	Maternal characteristics only maternal age, auto-immune disease, ART, HELLP/PE in a previous pregnancy, mutations in thrombophilia genes Full model Maternal characteristics + miR-1-3p, miR-17a-5p, miR-243a-3p, miR-146a-5p,miR-181a-5p, miR-499a-5p)	Development only Logistic regression	Maternal characteristics only AUC = 0.903, DR = 71.43%, 10% FPR Full model AUC = 0.979, DR = 85.71%, 10% FPR
Nan [44] 2023 Spain	Term PE; PE^@^ (GH (≥ 2 × SBP ≥ 140 mmHg and DBP ≥ 90 mmHg in ≥ 4 h, GA ≥ 20), with proteinuria ≥ 300 mg/24 h or 1 + on dipstick, or maternal organ dysfunction) ≥ GA37 and Pre-term PE; PE^@^ ≤ GA37	Singleton	Major anatomic malformations, confirmed chromosomal or genetic abnormalities, second-trimester pregnancy loss	Case–control	Not known	1,390 of which 51 with PE	‘Maternal factors’ (smoking, previous SGA, ART, MAP, and mean UAt-PI), PlGF, sFlt-1, uric acid, NT-ProBNP	1. Maternal factors; 2. Maternal factors + PlGF; 3. Maternal factors + sFlt-1; 4. Maternal factors + uric acid; 5. Maternal factors + NT-ProBNP; 6. Maternal factors + PlGF + sFlt-1 + uric acid + NT-ProBNP	Development only Multivariate analysis	1. AUC = 0.668 2. AUC = 0.710 3. AUC = 0.697 4. AUC = 0.581 5. AUC = 0.727 6. AUC = 0.770
Zhou [45] 2023 China	PE; hypertension ≥ GA20 with proteinuria or thrombocytopenia, impaired liver function, renal insufficiency, pulmonary edema, cerebral or visual disturbances Early onset PE, < GA34; Preterm PE < GA37	Han Chinese	pregestational diabetes, chronic hypertension, kidney related diseases, autoimmune diseases, smoking and alcohol during pregnancy, aneuploidies and major fetal abnormalities, termination, miscarriage, fetal death before < GA24	Cohort multicenter	Samples at 12–33	917 of which 202 with preterm PE	IVF, MAP, 7656 cfRNA molecules, including 4780 mRNAs, 239 lncRNAs, 493 miRNAs, 390 transfer RNAs, and 1754 other types of RNA	Preterm (AvNN) IVF, MAP, 13 cfRNA profiles (5 were mRNAs (ALB, FGA, LEP, IGFBP5) and 1 and 8 were miRNAs (MIR130A, MIR144, MIR19B1, MIR215, MIR376C, MIR27A, MIR106A and MIR33A)) Early-onset (SVM) IVF, MAP, 13 cfRNA profiles (7 were mRNA genes (ALB, FGA, IGFBP5, S100A9, APOA1, CGA, and SERPINA1), 1 was an lncRNA gene (MALAT1), and 5 were miRNA genes (MIR190A, MIR376C, MIR27A, MIR106A, and MIR215))	Development and validation Logistic regression (10 × cross-validation)	Preterm (AvNN) Development: AUC = 0.91 Validation 1: AUC = 0.81 Validation 2: AUC = 0.03 Early-onset (SVM) Development: AUC = 0.93 Validation 1: AUC = 0.88
Xue [46] 2023 China	Early-onset PE^¤^ (≥ 2 × SBP ≥ 140 mmHg and/or DBP ≥ 90 mmHg in ≥ 4 h, GA ≥ 20, with proteinuria (≥ 300 mg/24 h or ≥ 2 + on dipstick) or organ dysfunction, or liver involvement, or neurological complications or hematological complications) ≤ GA34	Singleton	Non-singleton, miscarriage, fetal death, intrauterine chromosomal disorders, fetal malformations, and missing laboratory data	Case control	6–10	800 of which 91 with PE	Clinical (12) Diabetes, thrombotic diseases, SLE, APS, kidney disease, ART, OSA, pre-pregnancy BMI, age > 35y, multiple pregnancies, primipara, history of eclampsia or PE Laboratory (38) ALB, ALT, ALP, complement C1q, Ca, Cr, CRP, GGT, GLB, TG, TC, HDL-C, LDL-C, Lp(a), ApoA1, ApoB, sdLDL-CB, TP, TBA, T-Bil, D-bil, UA, UREA, P, LYM, NEU, PLT, NEU/LTM ratio, PLT/LYM ratio, PT, PTA, APRR, FIB, D-Dimer, FDP, TT, Cystatin C, TCHO	Logistic regression Clinical Clinical + Laboratory Decision tree Clinical Clinical + Laboratory Support vector machine Clinical Clinical + Laboratory	Development only Logistic regression Support vector machine	AUC = 0.78 AUC = 0.86 AUC = 0.74 AUC = 0.77 AUC = 0.66 AUC = 0.93

^*^, definition of PE according to Magee et al. (2014)[59]; ^¥^, definition of PE according to Brown et al. (2001) [57]; ^α^, use of evolution agents, insemination, and/or assisted reproductive techniques; ^γ^, diabetes, hypertension, renal disease, antiphospholipid syndrome; ^δ^, adjusted for CRL; ^ε^, including ART;^ζ^, smoking 3 months before pregnancy, smoking at registration, snuff 3 months before pregnancy, snuff at registration, alcohol consumption 3 months before registration, alcohol consumption at registration; ^η^, To be considered at risk of PE, women had to meet one of the following inclusion criteria: PE, eclampsia, HELLP syndrome or IUGR in a previous pregnancy; pre-existing chronic hypertension without proteinuria; gestational hypertension (new-onset hypertension in pregnancy); pre-existing renal disease (kidney transplantation or creatinine clearance < 60 mL/min); pre-existing diabetes mellitus Type I (insulin dependent); mean uterine artery Doppler pulsatility index (UtA-PI) > 1.45 (at 19–20 weeks); thrombophilia (antiphospholipid syndrome, protein C deficiency, protein S deficiency, antithrombin deficiency, factor V Leiden mutation); multiple pregnancy; age ≥ 40 years and conceived with assisted reproductive technologies (ART). Women with two or more of the following risk factors were also included: nulliparity; body mass index ≥ 35 kg/m2; diastolic blood pressure > 80 mmHg at study inclusion; age ≥ 40 years; and family history (mother or sister) of PE, eclampsia or HELLP syndrome;

^&^, definition of PE according to Lindheimer et al. (2009) [60]; ^$^ definition of HELLP according to Mississippi classification [61] and Tennessee classification [62]; ^@^, definition of PE according to Brown et al. (2018) [63]; ^§^Gestational age according to Figueras [64]; ¤, definition of PE according to Poon et al. (2019), FIGO [65]; *CRL*, crown-rump-length; *UtA-PI*, uterine artery pulsatility index, *PAPP-A*, pregnancy-associated plasma protein-A; *PlGF*, placental growth factor; *sFlt-1*, soluble fms-like tyrosine kinase-1, *free β-HCG*, free Beta human chorionic gonadotropin; *AFP*, alpha-fetoprotein; *DR*, detection rate; *FPR*, false positive rate; *AUC*, area under the curve; *CI* confidence interval; *GA*, gestational age; *SGA*, small for gestational age; *SBP*, systolic blood pressure; *DBP*, diastolic blood pressure; *IPICC*, International Prediction of Pregnancy Complications Network; *CR*, competing risk; *MF*, maternal factors; *Hb*, hemoglobin; *FH*, family history; *HT*, hypertension; *UTI*, urinary tract infection; *CKD*, chronic kidney disease; *LMWH*, low molecular weight heparin; *DM*, diabetes mellitus; *GH*, gestational hypertension; *GWG*, gestational weight gain; *AC*, abdominal circumference; *ART*, assisted reproductive technique; *WBC*, white blood cell count; *ALT* alanine aminotransferase; *GGT*, γ-glutamyl transferase, *FPG*, fasting plasma glucose; *TBA*, total bile acid; *UN*, urea nitrogen; *UA*, uric acid; *WC*, waist circumference; *SLE*, systemic lupus erythematosus; *IVF*, in vitro fertilization; *MAP*, mean arterial pressure; *APS*, antiphospholipid syndrome; *OSA*, obstructive sleep apnea; *ALB*, albumin; *ALT*, alanine transaminase; *AST*, aspartate transaminase; *ALP*, alkaline phosphatase; *Ca*, calcium; *Cr*, creatinine; *CRP*, C-reactive protein; *GLB*, globulin; *TG*, triglyceride; *TC*, total cholesterol; *HDL-C*, high-density lipoprotein cholesterol; *LDL-C*, lowdensity lipoprotein cholesterol; *Lp (a)*, lipoprotein (a); *ApoA1*, apolipoprotein A1; *ApoB*, apolipoprotein B; *sdLDL-C*, small dense low density lipoprotein; *TP*, total protein; *TBA*, total bile acid; *T-Bil*, total bilirubin; *D-bil*, direct bilirubin; *UA*, uric acid; *P*, phosphorus, *LYM*, absolute value of lymphocyte; *NEU*, absolute value of neutrophil; *PLT*, platelet count; *PT*, prothrombin time; *PTA*, prothrombin activity; *APTT*, activated partial thrombin time; *FIB*, fibrinogen; *FDP*, fibrinogen degradation Products; *TT*, thrombin time; *PRS*, polygeneic risk score. ^€^, definition of PE by ACOG 2013 [87]; ^@^, definition of PE according to Brown et al. (2018) [63]; ^‰^, definition of GH and PE according to ISSHP (1988)[65]; *SG*, small for gestational age; ^¥^, definition of PE according to Brown et al. (2001) [66]; ^¶^, definition of PE by Norwegian Association of Obstetricians and Gynaecologists (2013); *SLE*, systemic lupus erythematosus; *DM*, diabetes mellitus; *IVF*, in vitro fertilization; *MAP*, mean arterial pressure; *PAPP-A*, pregnancy associated plasma protein A; *PlGF*, placental growth factor, *UtA-PI*, uterine artery pulsatility index; *DC*, dichorial twin; *MC*, monochorial twin; *results from regresions fitted to twin data

Some studies applied machine learning such as random forest [37, 40], XGBoost [40] or support vector machines [40] to build prediction models.

### Definition of Outcome

Definitions of PE varied among studies. While most studies defined PE as hypertension with proteinuria [37, 40, 56], several studies followed definitions based on different guidelines: Brown et al. (2001) [34, 35, 37, 49, 50, 55, 66], ISSHP (1988) [54, 67], Poon et al. (2019), FIGO [38, 68], Magee et al. (2014) [39, 59], and Brown et al. (2018) [44, 63]. The study by Hromadnikova [43] specified HELLP as primary outcome, following the Mississippi and Tennessee classifications [61, 62]. For participant definitions, some studies [31, 42, 43, 46, 51] provided only a limited definition of eligibility criteria, with little to no description of the study population.

Other studies developed competing risk models based on the hypothesis that PE can arise either before or after delivery. It is assumed that if the pregnancy were to continue indefinitely, all women would develop PE, resulting in a competition between the timing of delivery – whether it occurs before or after the onset of the condition. Consequently, some models used gestational age (GA) at the onset of PE as primary outcome [32, 34, 35, 48].

Timing at which the prediction model is intended to be used varied between studies and was not always clearly described [37, 40, 42, 44]. Most models were intended to be used at the end of the first trimester (GA 10–11 weeks) or the beginning of the second trimester (GA 12–13 weeks) [31, 34–36, 38, 39, 43, 47–53, 55, 69]. Models presented in two studies [41, 46] were intended for early first trimester. One model was intended for use between GA16-20 weeks [33], and one for 19–23 weeks [32]. One study published multiple models for different timepoints (GA 19–20, 23–24, 27–28) [56].

### Predictors

Predictors included clinical parameters, biomarkers or a combination of both. Most commonly used biomarker predictors were Uterine Artery Pulsatility Index (UtA-PI), mean arterial pressure (MAP), placental growth factor (Pl-GF), pregnancy associated plasma protein A (PAPP-A). ART definition varied among different studies.

Bayesian models calculated the *a-priori* risk based on selected ‘maternal factors’, which – after clarification from the authors – consistently included maternal age, weight, height, racial origin, interpregnancy interval, GA at delivery of previous pregnancy with and without PE, method of conception, chronic hypertension, diabetes mellitus (DM), systemic lupus erythematosus (SLE)/anti-phospholipid syndrome (APS), dichorionic and monochorionic twins, and MoM biomarker values [31, 32, 34, 35] [58, 69]. These biomarker measurements were standardized to multiples of the expected normal mean (MoM) [70], adjusted for factors such as maternal age, BMI, smoking and racial origin. In these studies, method of conception was included in the *a-priori* risk assessment. After author inquiry for nine studies, method of conception was categorized in spontaneous, ovulation induction and IVF [31, 32, 34, 35, 47–50, 52, 53]. One study defined ART as conception by IVF (yes/no) [36]. For two studies ART definition could not be retrieved [33, 51].

In most frequentist models, ART was not specified further than requiring ART (yes/no) [37–40, 43, 44, 46, 52, 56]. In two models, ART was defined as requiring IVF (yes/no) [42, 45]. In two studies, ART was categorized in spontaneous, requiring IVF or ovulation induction [41, 54]. For categorical variables, details on coding were not specified (e.g. categorization or use of dummy variables).

Only seven out of 26 studies described the variable selection and model building process in detail [37, 38, 40, 45, 46, 52, 55]. Only three out of 26 papers provide the full model in their paper [48, 54, 55] with individual coefficients. Reporting of unadjusted associations between predictor and outcome was also limited [31–33, 35, 36, 38, 44, 56]. Only one study by Lewandowska et al. [38] reported the effect of ART on model performance. Here, performance did not improve by including ART to the model.

### Performance, Generalizability, and Applicability

Due to substantial heterogeneity in study populations, predictors and outcome definitions, a meta-analysis was not performed. Instead, performance, generalizability and applicability are presented narratively. Performance of all included models is summarized in Tables 1 and 2. Model performance with regard to discrimination, including receiver operating curves and c-statistic values with 95% confidence intervals (CIs) was provided in all studies. However, calibration information was limited. One study used Hosmer–Lemeshow test p-values to support model fit to observed outcomes [44], while another briefly mentioned calibration, but without supporting statistics [42]. O’Gorman et al. (2017) [53] and Hromadnikova et al. (2023) [43] reported highest AUCs (Tables 1 and 2). Model performance of Bayesian models improved with addition of biomarkers to maternal factors alone [31, 32, 34–36, 48, 49, 52, 53].

None of the studies presented prediction models as online calculators for health professionals or patients. The majority were ‘development only’ studies [32, 33, 35, 37–39, 41–44, 47–49, 54–56], with a few exceptions providing external validation. Park et al. (2013) [50] provided external validation of Poon et al. (2009) [47], Skråstad et al. (2015) [51] validated Wright et al. (2012) [48], O’Gorman et al. (2017) [53] validated Akolekar et al. (2013) [71]. The latter was not included in this review as it was omitted from de Kat et al.’s initial selection. After correspondence with the author [13], it was clarified that the exclusion was due to an incomplete description of the study population and an unclear overlap between populations of different studies. Fransisco et al. (2017) [34] validated Francisco et al. (2017) [35]. Of the validation studies (n = 7), only Zhou et al. [45] showed a comparison with the development data of the distribution of predictors and outcome.

### Risk of Bias Assessment

The PROBAST tool comprises the following domains: participant selection, predictor assessment, outcome determination, and analytical methods [18]. Results for all included studies are summarized in Table 3. Many studies exhibited a moderate risk of bias, primarily due to analytical issues. For instance, some studies excluded patients with missing outcome data without motivation [31, 33, 38, 40, 41, 46, 47, 50]. Additionally, some studies reported a limited number of events per variable, raising the risk of model overfitting [31, 34, 35, 40, 41, 44–46, 50, 51, 56]. Concerns about applicability also arose, particularly where predictor definitions were unclear and required author correspondence for clarification of ‘maternal factors’ [31–36, 47, 48, 50–53]. Some studies lacked adequate outcome definitions [42, 43], and it was often unclear whether data-driven categorization was avoided or non-linearity of predictors had been addressed. Detailed overview of domain specific assessments and derivation of the overall ratings are provided in *Supplement 2*.

**Table 3:** PROBAST scores for the studies included

	Risk of bias (ROB)				Applicability			Overall	
	Participants	Predictors	Outcome	Analysis	Participants	Predictors	Outcome	ROB	Applicability
**Bayesian models**									
Poon 2009 [47]	+	+	+	-	+	-	+	?	+
Wright 2012 [48]	+	+	+	-	+	-	+	?	+
Khalil 2012 [49]	?	?	+	-	+	+	+	?	+
Park 2013 [50]	?	+	+	-	+	+	+	?	+
Skråstad 2015 [51]	+	+	+	-	+	+	-	?	?
O’Gorman 2016 [52]	+	+	+	-	+	?	-	?	?
O’Gorman 2017 [53]	+	+	+	-	+	+	?	?	+
Maymon 2017 [33]	+	?	+	-	+	+	+	?	+
Francisco 2017 [35]	+	+	+	-	-	+	?	?	?
Francisco 2017 [34]	+	+	+	-	+	-	-	?	-
Sapantzoglou 2021 [32]	+	+	+	-	+	+	+	?	+
Chaiyasit 2022 [31]	-	?	?	-	-	?	+	-	?
Moungmaithong 2023 [36]	+	+	+	-	+	-	?	?	?
**Frequentist models**									
Poon 2010 [54]	?	+	+	-	+	+	+	?	+
Wright 2015 [55]	+	+	+	-	+	+	?	+	+
Perales 2017 [56]	+	?	?	-	+	+	?	?	?
Sandström 2019 [37]	+	+	+	?	+	+	?	+	+
Lewandowska 2020 [38]	+	+	?	-	?	+	?	?	?
Boutin 2021 [39]	+	+	+	-	+	+	+	?	+
Li 2021 [40]	+	?	+	?	+	?	+	?	?
Pétursdóttir 2022 [41]	?	+	+	-	+	+	+	?	+
Nurkkala 2023 [42]	?	?	?	?	-	?	+	?	?
Hromadnikova 2023 [43]	-	?	?	-	-	?	+	-	?
Nan 2023 [44]	+	?	+	-	+	?	+	?	?
Zhou 2023 [45]	-	?	?	-	?	+	+	-	?
Xue 2023 [46]	?	?	?	-	+	+	+	?	+

+ indicates low risk of bias/low concern regarding applicability;—indicates high risk of bias/high concern regarding applicability; and? indicates unclear risk of bias/unclear concern regarding applicability

#### Quality of Reporting

Regarding reporting quality, each study was assessed using the TRIPOD checklist, with individual scores available in Supplement 3. Most studies were considered development-only [32, 33, 35, 37–39, 41–44, 46–49, 52, 54–56], hence nine subitems of the TRIPOD checklist were deemed non-applicable for these studies [23]. Additionally, as no therapeutic studies were included, the sub-item related to medication use was also non-applicable, for all studies. Therefore, the final quality of reporting score was calculated as the number of items met divided by the total number of scorable items. For development-only studies, the total number of items was 27, while for validation studies, the total number was 36. The percentage of TRIPOD checklist items met for each of the 26 included studies is shown in Table 4, ranging from 41% [43] to 89% [37]. The most frequently missing items included the identification of the study type in the title, blinding in outcome and predictor assessment, clarification of sample size, handling of missing data, handling of predictors in the analysis, description of model updating, identification of differences between the development and validation sets, comparison between these sets, presentation of the full model including coefficients, and providing context for the model’s use and clinical implications.

**Table 4:** TRIPOD scores for the studies included

Bayesian models	Publication type	Items met	Items not met	Score
Poon 2009 [47]	Development only	15	12	56%
Wright 2012 [48]	Development only	18	9	67%
Khalil 2012 [49]	Development only	19	8	70%
Park 2013 [50]	Validation only	20	16	55%
Skråstad 2015 [51]	Validation only	20	16	55%
O’Gorman 2016 [52]	Development only	17	10	64%
O’Gorman 2017 [53]	Validation only	16	19	46%
Maymon 2017 [33]	Development only	17	10	63%
Francisco 2017 [35]	Development only	17	10	63%
Francisco 2017 [34]	Development only	16	11	59%
Sapantzoglou 2021 [32]	Development only	18	9	67%
Chaiyasit 2022 [31]	Development and validation	19	17	53%
Moungmaithong 2023 [36]	Development and validation	22	14	61%
**Frequentist models**				
Poon 2010 [54]	Development only	17	10	63%
Wright 2015 [55]	Development only	20	7	74%
Perales 2017 [56]	Development only	20	7	74%
Sandström 2019 [37]	Development only	24	3	89%
Lewandowska 2020 [38]	Development only	22	5	81%
Boutin 2021 [39]	Development only	18	9	67%
Li 2021 [40]	Development and validation	25	11	69%
Pétursdóttir 2022 [41]	Development only	17	10	63%
Nurkkala 2023 [42]	Development only	15	12	56%
Hromadnikova 2023 [43]	Development only	11	16	41%
Nan 2023 [44]	Development only	18	9	67%
Zhou 2023 [45]	Development and validation	17	19	47%
Xue 2023 [46]	Development only	16	11	59%

The quality of reporting score was calculated as the number of items met divided by the total number of scorable items. For development-only studies, the total number of items was 27, while for validation studies, the total number was 36

## Discussion

This systematic review highlights the challenges in accurately predicting PE in ART pregnancies. Despite the well-established link between ART and increased PE risk [2–4], prediction models for PE rarely include ART as a predictor. We identified 231 prediction models in literature (161 records identified from Medline from 2017, 70 from the review by De Kat et al.), with none of the models specifically developed for the ART population. In the absence of these studies, we must rely on models that merely include ART as a predictor. We observed that in the 231 identified studies, only a remarkably small proportion of 26 included ART as a predictor. As previously noted by De Kat et al. (2019) [13], the quality of these prediction studies for PE varies widely, with few models fully available, let alone be externally validated or implemented in clinical practice [71]. With the introduction of PROBAST and the TRIPOD checklist to improve prediction model development and reporting, we expected higher-quality studies after the review by De Kat et al. in 2019 [13].

We identified several concerns regarding the methodological quality of the included studies. One significant issue is the inconsistency in definitions of PE across studies. While some of this variation can be attributed to historical shifts in clinical guidelines, many studies deviate from the contemporary definition of PE. Currently, PE is defined according to the ISSHP 2018 guideline, as gestational hypertension accompanied by ≥ 1 of the following new-onset conditions at or after 20 weeks’ gestation: proteinuria, other maternal organ dysfunction, including: acute kidney insufficiency, liver involvement with or without right upper quadrant or epigastric abdominal pain, neurological complications or hematological complications [7, 59]. The lack of outcome standardization complicates the comparability and generalizability of studies and emphasizes the need for a consistent approach to outcome definitions in future model development.

In addition, many studies had insufficient events per variable, which increases the risk of model overfitting and limits generalizability. To develop robust prediction models for binary outcomes like PE ‘yes’ or ‘no’ or time-to-event outcomes such as gestational age at PE onset, a minimum of 10 (preferably 20) events per predictor is often recommended [19]. When developing a prediction model, accurate estimation of predictor effects is crucial and can be supported by choosing the sample size taking the outcome proportion and expected performance into account [72].

In our study, we included both frequentist and Bayesian prediction models. Bayesian models incorporate prior knowledge, and use this knowledge to specify prior probabilities. These priors are then updated with new available data [73], whereas frequentist models calculate predictions solely based on the data at hand. This distinction makes direct comparison between studies using Bayesian and frequentist models challenging. In this review, studies using Bayesian models often lacked specification of maternal factors and standardization of biomarker values into multiples of the mean (MoM), with adjustments for unspecified maternal characteristics. These maternal characteristics were often used as priors, but the composition of these prior and their impact on the resulting models were not well defined. Additionally, it was unclear whether these models followed a two-step or single-step approach. Although we contacted authors of models included in our study to clarify the maternal factors and whether ART was incorporated, this lack of clarity impedes reproducibility of these studies.

Moreover, most studies (19/26) lack external validation, making interpretation of their generalizability difficult. It is widely recommended that a prediction model should not be used in clinical practice before being externally validated in other individuals than those in the training set [74–78]. Finally, proper handling of continuous and categorical variables also varied widely, which may contribute to inaccurate predictions in practice.

We also found significant reporting deficiencies in the included studies. For instance, only seven out of 26 studies detailed their variable selection and model building process. While all studies that specified candidate predictors included ART in the final model, only three reported full models with coefficients available. Reporting of unadjusted predictor-outcome associations was also limited.

Interestingly, the models with highest performance [43, 53] had the lowest reporting scores (Table 4). For these models, it remains unclear whether blinding of outcome and predictor assessment took place, how predictions were calculated, how the model was built, how missing data were handled, and how the prediction model should be used. These limitations in reporting compromise the transparency and reproducibility of the models, potentially hindering their accurate interpretation and appropriate application in clinical practice [77, 79]. Despite the TRIPOD statement [20] publication in 2015, reporting quality seems not improved over time. These findings underscore the need for future prediction studies to enhance transparency and compliance with the TRIPOD statement.

Our systematic review has some limitations. First, we relied exclusively on one database, MEDLINE, for study collection, which may increase the risk of selection bias. Although we screened over 15,000 articles, we acknowledge that due to absence of additional databases such as Embase, Cochrane Library, Web of Science, Scopus and grey literature sources, relevant studies may have been missed. Notably, this approach is consistent with that of De Kat et al. [13], who also relied on MEDLINE as their only database.

Furthermore, the extensive screening and appraisal process is resource intensive which resulted in a time gap between completion of the search (July 31 st 2023) and manuscript finalization. As a result, more recent prediction models may have been published after the search end date, that were not included in this review.

Additionally, screening over 15,000 articles raises the potential for error during the selection process. Moreover, our search strategy was based on the approach used by De Kat et al. (2019) [13], but during data extraction, we identified some key studies such as Akolekar et al. [70] and Loughna et al. [80], which were development studies of prediction models that were later validated by O’Gorman et al. (2016) [52] and Skråstad et al. (2015) [51] respectively, and had been omitted by the authors [13]. Also, De Kat et al. included only papers with AUC > 0.6, a threshold which after correspondence with the authors [13] had been pragmatically chosen to ensure clinical relevance and keep the project manageable. This could also have contributed to the absence of abovementioned papers.

For our review,, we included only models in which ART was retained in the final model. Consequently, we did not capture models that initially considered ART as a candidate predictor but later excluded it through backward selection or LASSO. For most prediction models, assessing the effect of ART on performance was impossible, as it was embedded within maternal factors (Bayesian models) without detailed reporting.

Besides, model development and full coefficients were not provided (Supplement 3).

Finally, this systematic review does not assess whether the externally validated prediction models were presented as risk calculator, web tool or implemented in electronic health records.

Our study also has notable strengths, including it being the first review to comprehensively identify prediction models for PE that incorporate ART as a predictor. As we could not endorse a specific model to capture PE risk for ART pregnancies specifically, this study highlights the important gap in current risk assessment strategies for PE in ART pregnancies. The increased PE risk following ART likely reflects both underlying maternal reproductive characteristics and ART specific mechanisms, including programmed embryo transfer without a corpus luteum, multiple gestations, and altered maternal–fetal immune interactions in OD pregnancies [1, 3, 4, 8, 10, 12]. Omitting ART from prediction models may therefore compromise risk assessment in ART pregnancies. Remarkably, models that included ART as a predictor in our study generally treated it as a single entity and rarely distinguished between IVF, ICSI, frozen/thawed embryo transfer and OD, despite substantial variation in PE risk across these treatment modalities. For example, the risk of hypertensive complications in OD is more than doubled compared to conventional IVF and ICSI [11, 81–85]. Embryo donation, or double donation (DD), encompasses possibly an even higher risk due to the separate risk associated with sperm donation [60]. This study therefore provides an important starting point for future research.

Other strengths of this study are the extensive screening of over 15,000 studies, and adherence to the TRIPOD statement, and the use of PROBAST for accurate quality assessment of prediction models.

Future research should focus on developing and externally validating prediction models tailored to ART. In addition, we recommend future prediction models including ART as predictor to disaggregate ART types, such as OD and DD. Improved risk assessment could aid in disease management, support periconceptional counselling, and help minimize donor exposure to the risk associated with oocyte retrieval [86]. Understanding hypertensive risk in ART pregnancies could improve early recognition and insights into PE pathophysiology. Given the poor reproducibility and lack of external validation in these populations, we aim to eventually develop a dedicated prediction model for PE in OD/DD pregnancies, in accordance with to PROBAST and TRIPOD guidelines [64].

## Conclusion

This systematic review illustrates the limited availability of robust, ART-specific models that predict the development of PE. Limitations in current studies, including inconsistencies in outcome definitions, insufficient events per variable, lacking external validation, varying approaches to handling continuous and categorical variables and limited reporting quality complicate PE risk assessment in the ART population. Based on current findings, we are unable to endorse any prediction model for clinical practice in the ART population at this time.

Future studies should focus on creating a high-quality prediction model, following PROBAST and TRIPOD guidelines, that incorporates ART as a predictor for PE. Ideally, this model should be developed and validated within ART populations specifically, to improve its accuracy for this high risk group.

## Supplementary Information

Below is the link to the electronic supplementary material.

Supplementary file1 (DOCX 3176 KB)

## Data Availability

No additional datasets were generated for this systematic review. All data used in this review are reported in this article and the supplementary materials, including the detailed scoring analyses, with the corresponding sources provided in the reference list.

## Declarations

### PROSPERO Registration

This review was registered in PROSPERO (ID CRD420250477748) on the 11th of March 2025.
