# Supplementary material for: Assisted Reproductive Technology as Predictor for Preeclampsia: A Systematic Review of Current Clinical Prediction Models
Source: Reprod Sci. 2026 Sep 15;33(10):1907–30. doi: 10.1007/s43032-026-02168-7 (PMC13645924; doi:10.1007/s43032-026-02168-7)
Supplement: Supplementary file 1 [file 43032_2026_2168_MOESM1_ESM.docx]

**Search Strategy – adapted from De Kat et al. (2019)**

Search terms (search 10-1-2017):

1. Validat*[tiab] OR Predict*[ti] OR Rule*[tiab]

2. Predict*[tiab] AND (Outcome*[tiab] OR Risk*[tiab] OR Model*[tiab])

3. (History[tiab] OR Variable*[tiab] OR Criteria[tiab] OR Scor*[tiab] OR Characteristic*[tiab] OR Finding*[tiab] OR Factor*[tiab]) AND (Predict*[tiab]

OR Model*[tiab] OR Decision*[tiab] OR Identif*[tiab] OR Prognos*[tiab])

4. Decision*[tiab] AND (Model*[tiab] OR Clinical*[tiab] OR Logistic Model*[tiab])

5. Prognostic[tiab] AND (History[tiab] OR Variable*[tiab] OR Criteria[tiab] OR Scor*[tiab] OR Characteristic*[tiab] OR Finding*[tiab] OR Factor*

[tiab] OR Model*[tiab])

6. “risk score”[All fields] OR “prediction model”[All fields] OR “prediction rule”[All fields] OR “risk assessment”[All fields] OR “algorithm”[All

fields]

7. # 1 OR #2 OR #3 OR #4 OR #5 OR #6

8. (pregnan*[tiab] OR obstetric*[tiab] OR woman[tiab] OR women[tiab]) AND

(abruption [tiab] OR growth restrict* OR preeclampsia[tiab] OR pre-eclampsia [tiab] OR pregnancy induced hypertension[tiab] OR HELLP[tiab] OR preterm deliver* [tiab] OR stillbirth OR “small-for-gestational-age” OR preterm birth [tiab] OR prematurity [tiab] OR “fetal death” [tiab] OR “perinatal mortality” [tiab])

9. #7 AND #8

31-07-2023

N = 15,305
